# Orphan G-Protein Coupled Receptor GPRC5B Is Critical for Lymphatic Development

**DOI:** 10.3390/ijms23105712

**Published:** 2022-05-20

**Authors:** Wenjing Xu, Nathan P. Nelson-Maney, László Bálint, Hyouk-Bum Kwon, Reema B. Davis, Danielle C. M. Dy, James M. Dunleavey, Brad St. Croix, Kathleen M. Caron

**Affiliations:** 1 Department of Cell Biology and Physiology, The University of North Carolina, Chapel Hill, NC 27599, USA; xwj1984@gmail.com (W.X.); nathan_nelson-maney@med.unc.edu (N.P.N.-M.); laszlo_balint@med.unc.edu (L.B.); hyoukbum.kwon@gmail.com (H.-B.K.); reemabiswas@gmail.com (R.B.D.); ddy3@live.unc.edu (D.C.M.D.); 2 Tumor Angiogenesis Unit, Mouse Cancer Genetics Program, National Cancer Institute–Frederick, NIH, Frederick, MD 21702, USA; jmdunleavey@gmail.com (J.M.D.); stcroixb@mail.nih.gov (B.S.C.)

**Keywords:** orphan G protein-coupled receptors, lymphatic, GPRC5B, lymphangiogenesis, edema, cardiac lymphatics, growth restriction

## Abstract

Numerous studies have focused on the molecular signaling pathways that govern the development and growth of lymphatics in the hopes of elucidating promising druggable targets. G protein-coupled receptors (GPCRs) are currently the largest family of membrane receptors targeted by FDA-approved drugs, but there remain many unexplored receptors, including orphan GPCRs with no known biological ligand or physiological function. Thus, we sought to illuminate the cadre of GPCRs expressed at high levels in lymphatic endothelial cells and identified four orphan receptors: GPRC5B, AGDRF5/GPR116, FZD8 and GPR61. Compared to blood endothelial cells, GPRC5B is the most abundant GPCR expressed in cultured human lymphatic endothelial cells (LECs), and in situ RNAscope shows high mRNA levels in lymphatics of mice. Using genetic engineering approaches in both zebrafish and mice, we characterized the function of GPRC5B in lymphatic development. Morphant *gprc5b* zebrafish exhibited failure of thoracic duct formation, and *Gprc5b^−/−^* mice suffered from embryonic hydrops fetalis and hemorrhage associated with subcutaneous edema and blood-filled lymphatic vessels. Compared to *Gprc5^+/+^* littermate controls, *Gprc5b^−/−^* embryos exhibited attenuated developmental lymphangiogenesis. During the postnatal period, ~30% of *Gprc5b^−/−^* mice were growth-restricted or died prior to weaning, with associated attenuation of postnatal cardiac lymphatic growth. In cultured human primary LECs, expression of GPRC5B is required to maintain cell proliferation and viability. Collectively, we identify a novel role for the lymphatic-enriched orphan GPRC5B receptor in lymphangiogenesis of fish, mice and human cells. Elucidating the roles of orphan GPCRs in lymphatics provides new avenues for discovery of druggable targets to treat lymphatic-related conditions such as lymphedema and cancer.

## 1. Introduction

The lymphatic system is a complex vascular network that permeates nearly every organ and plays critical roles in the maintenance of fluid homeostasis, the trafficking and maturation of immune cells and the absorption of intestinal lipids, nutrients and hormones [1,2]. In recent years, the field has come to appreciate that, just like the blood vascular system, the lymphatic system is comprised of anatomically and molecularly heterogeneous vessels that execute different functions depending on their developmental origin or their organ-specific functions [2,3]. The pathogeneses of cancer, cardiovascular disease, and autoimmune diseases, some of the most prevalent diseases, are often linked to lymphatic defects and can result in lymphedema [4,5,6]. An estimated 250 million individuals worldwide suffer from lymphedema, and yet the only recommended therapies from the International Society of Lymphology are compression garments and manual massage [7]. There are currently few, if any, feasible pharmacological targets for the specific and effective therapeutic modulation of lymphatic vessels [8,9]. Thus, the discovery of pharmacologically tractable factors that preferentially and specifically control and modulate the functions of lymphatics remains a high priority.

When considering the different classes of proteins encoded by the human genome that are predicted to be pharmacologically tractable, nearly one quarter represent G protein-coupled receptors (GPCRs) [10,11], accounting for approximately 30–40% of all prescriptions worldwide [12]. Among the ~800 human GPCRs, over 100 remain either orphans (with no known ligand) or uncharacterized (with no clear physiological function), resulting in large-scale consortia dedicated to illuminating new pharmacological targets [13,14]. A very small number of GPCRs have been identified as therapeutic targets for lymphedema (based on the Target Importance and Novelty eXplorer (TIN-X) [15]), including CALCRL [16,17,18], CELSR1 [19], LPAR1/S1PR1 [20] and APJ [21]. Thus, a notable barrier in the field of lymphatic biology is the dearth of GPCRs, both known and orphan, that are specifically—or even preferentially—expressed in lymphatic endothelial cells (LECs) [22].

It is widely appreciated that acute regulation of most GPCRs occurs via cellular trafficking, G-proteins and desensitization, such that their gene expression levels are not only low compared to most cellular mRNAs but are also often under-represented in genome-wide expression analyses. Therefore, we made use of a pathway-focused RT-PCR array platform to probe for the enrichment of 380 different GPCRs expressed in hLECs compared to human umbilical venous endothelial cells (HUVECs), validating previously identified lymphatic GPCRs and illuminating several novel orphan GPCRs. We further used in vitro and in vivo animal models to reveal a critical function for the most enriched lymphatic GPCR, GPRC5B (GPCR class C group 5 member B) in lymphatic proliferation and development. There is a dearth of published data regarding GPRC5B.

## 2. Results

### 2.1. Lymphatic Endothelial Cell-Enriched GPCRs

To identify GPCRs with high expression in lymphatic endothelial cells, we performed a pathway-focused TaqMan microfluidic array on RNA isolated from primary human dermal lymphatic endothelial cells (LECs) and compared the expression levels to those obtained from primary human umbilical venous endothelial cells (HUVECs). A ranked list of the GPCRs significantly enriched in LECs compared to HUVECs is provided in Table 1. Amongst the top five most enriched genes, we were encouraged to identify *EDG1/SIP1R* (sphingosine 1-phosphate receptor) and *SSTR5* (somatostatin receptor), which have previously established roles in lymphatics [23,24,25], stimulating confidence in the success of our comparative approach. We were also intrigued by the fact that three of the top five receptors, and four total receptors, are characterized as orphan receptors, having no known endogenous ligand. These receptors include *GPRC5B*, *ADGRF5* (aka *GPR116*, Adhesion G Protein-Coupled Receptor F5), *FZD8* (Frizzled Class Receptor 8) and *GPR61* (G Protein-Coupled Receptor 61).

### 2.2. In Situ Localization of Lymphatic Endothelial Cell-Enriched GPCR Expression

**Figure 1:**
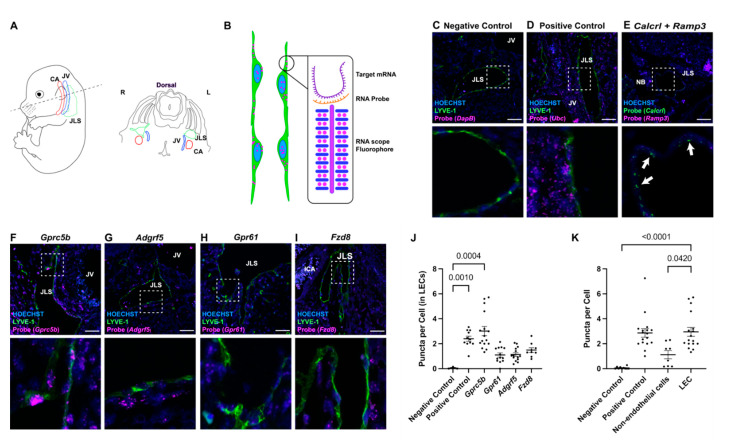
In situ localization of lymphatic endothelial cell-enriched GPCR expression. (**A**) Schematic diagram of E14.5 transverse section of mouse embryo depicting the sterotypic positioning of carotid artery (CA), jugular vein (JV) and jugular lymph sac (JLS). (**B**) Schematic diagram of RNAscope probe design, illustrating how a single mRNA molecule is amplified into a visible fluorophore puncta within a cell. (**C**–**I**) RNAscope images for indicated probes (magenta), Hoechst nulcei (blue) and the lymphatic maker LYVE-1 or *Calcrl* in panel (**E**) (green). Probe names are listed at top of images. Lower images are higher magnification views of the white dashed inset. White arrows in (**E**) point to lymphatic endothelial cells with double-labeling of the lymphatic GPCR, *Calcrl* (green) and *Ramp3* (magenta). Scale bars = 50 µm. (**J**) RNAscope quantification for each orphan receptor probe in lymphatic endothelial cells and (**K**) RNAscope quantification for *Gprc5b* in various tissue types. Puncta per cell is a relative measure of the number of mRNA transcripts for the target gene. Non-endothelial cells (non-ECs) represent all background cells. LEC = lymphatic endothelial cells. Significance calculated using Kruskal–Wallis nonparametric analysis of variance with Dunn’s multiple comparisons test. *p*-values equal to or less than 0.05 are shown.

To confirm the results of the comparative microarray analysis, we performed RNAscope on developing jugular lymph sacs (JLSs) in wildtype C57BL/6J mice at embryonic day 14.5 (E14.5) for each of the aforementioned orphan GPCRs. As depicted in Figure 1A,B, this spatiotemporal region was selected due to the well-established anatomical localization of nascent lymphatic vessels juxtaposed to the jugular vein (JV) and carotid artery (ICA). Combining RNAscope with traditional immunofluorescence for lymphatic vessel endothelial hyaluronan receptor (LYVE-1), a lymphatic endothelial cell marker, allows for the identification of the jugular lymph sac (JLS). As expected, we observed little to no background puncta for the negative control probe *DapB*, a gene not encoded in the eukaryotic genome (Figure 1C), and ubiquitous puncta signals for the *Ubc* positive control (Figure 1D). Furthermore, we confirmed the efficacy of RNAscope by visualizing the expression of the well-established lymphatic-related GPCR, *Calcrl* and its chaperone protein, *Ramp3*, in the JLS (Figure 1E).

Consistent with the microarray analysis, we observed very high levels of *Gprc5b* puncta in the LYVE-1-positive JLS, equivalent to the levels of the *Ubc* positive control, and with no discernible puncta in venous endothelial cells of the jugular vein (JV) (Figure 1F). This was quantified as fluorescent puncta per cell, with each puncta representing 1 or more mRNA molecules (Figure 1J). Similarly, we observed robust levels of *Adgrf5/Gpr116* puncta in the LYVE-1-positive JLS, though there was also evidence of expression within the jugular vein (Figure 1G).

*Gpr61* and *Fzd8* showed little to no puncta in LYVE-1-positive cells, despite showing expression in non-endothelial cells (Figure 1H,I). Quantitative analysis of puncta per cell confirmed the abundance of *Gprc5b* in LYVE-1-positive LECs of the JLS (Figure 1J), and revealed that Gprc5b expression is significantly higher in LECs than in non-endothelial cells (Figure 1K). Collectively, these data validate the in vivo expression of *Gprc5b* and *Adgrf5/Gpr116* in the developing lymphatic vasculature, with a significant enrichment for Gprc5b expression in lymphatic compared to non-endothelial cells.

### 2.3. Gprc5b Is Required for Lymphatic Development in Zebrafish

**Figure 2:**
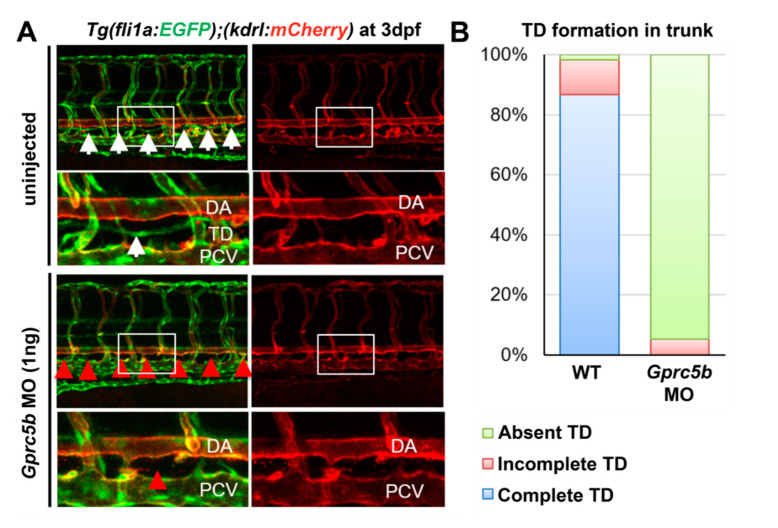
Zebrafish *gprc5b* morphant embryos exhibit decreased lymphatic vessel formation. (**A**) Confocal images of *Tg(fli1a: EGFP); Tg(kdrl: Hsa.HRASmCherry)* control (uninjected) and 1 ng of *gprc5ba* morpholino-injected larvae at 72 h post-fertilization (hpf). The boxes in the above panels are enlarged in the bottom panels, respectively. White arrows point to EGFP-positive TD in the trunk region. Red arrows point to defects in TD formation in the trunk region. Anterior to the left, dorsal to the top. DA, dorsal aorta; PCV, posterior cardinal vein; TD, thoracic duct. (**B**) Quantification of EGFP-positive/kdrl-negative thoracic duct in the trunk (six somites). Uninjected embryos (*N* = 10) and gprc5ba MO-injected larvae (*N* = 13), from three independent clutches.

Considering the high expression level of *Gprc5b* in developing lymphatics, we sought to determine whether *gprc5b* expression influences the growth and development of lymphatic vessels at an organismal level. Leveraging the tractability of systemic knock downs using morpholino constructs in zebrafish, we were able to readily assess whether *gprc5b* had an impact on developing zebrafish thoracic ducts. One ng of morpholinos (MOs) for *gprc5b* was injected into one-cell stage *Tg(fli1a:EGFP);Tg(kdrl:mCherry)* double-transgenic zebrafish embryos, permitting the visualization of vascular development in the trunk region. At 72 h post-fertilization (hpf) uninjected wildtype larvae displayed prototypic posterior vascular development with the *kdrl:mCherry*-positive dorsal aorta (DA) and doubly positive *fli1a:EGFP/kdrl:mCherry* posterior cardinal vein (PCV) separated by the emergence of a continuous *fli1a:EGFP-positive/kdrl:mCherry*-negative lymphatic thoracic duct (TD) in more than 80% of animals (Figure 2A, white arrows). However, there was a marked failure of lymphatic thoracic duct formation in *gprc5b* morphants (Figure 2A, red arrows), with 94.8% absence of TD (N = 13 *Gprc5b* morphant larvae) compared to only 1.6% absence of TD in uninjected embryos (N = 10 larvae) (Figure 2A,B). These data demonstrate that the expression of *gprc5b* is required for normal lymphatic development in zebrafish.

### 2.4. Lymphatic Development Is Attenuated in Murine Gprc5b^−/−^ Embryos

**Figure 3:**
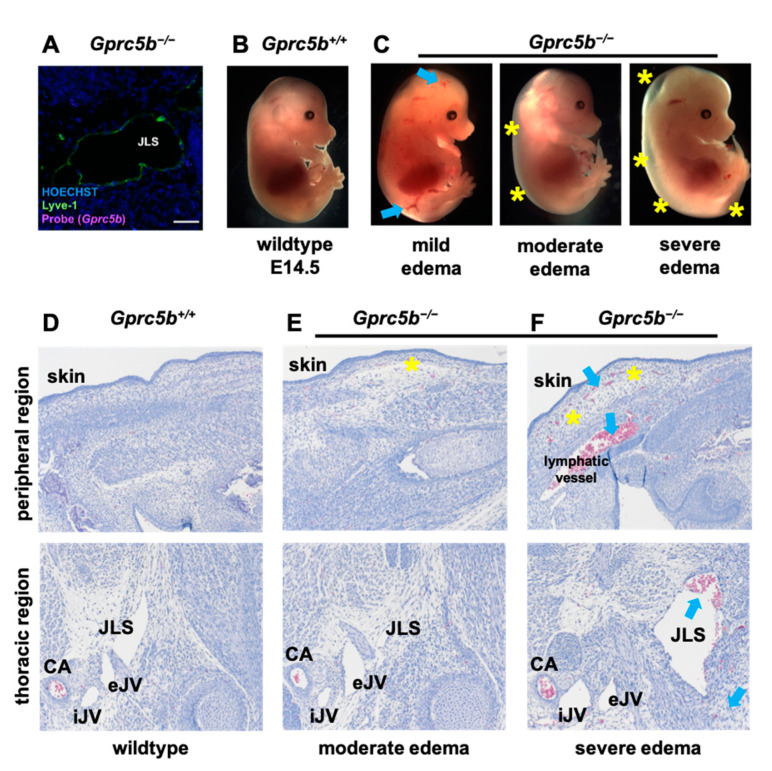
Lymphatic development is attenuated in murine *Gprc5b^−/−^* embryos. (**A**) RNAscope performed on jugular section of *Gprc5b^−/−^* embryo, confirming absence of mRNA expression (compare to Figure 1F): *Gprc5b* probe (magenta), LYVE-1 lymphatic marker (green), Hoechst cell nuclei (blue). (**B**) E14.5 *Gprc5b^+/+^* littermate control embryo. (**C**) E14.5 *Gprc5b^−/−^* embryos show graded levels of edema (yellow asterisks) and hemorrhagic plaques (blue arrows). Compared to normal *Gprc5b^+/+^* littermate controls (**D**), representative H&E images showing subcutaneous edema in the back skin (upper panels, yellow asterisks) and blood-filled lymphatic vessels (lower panels, blue arrows) in *Gprc5b^−/−^* embryos with moderate to severe edema (**E**,**F**). CA, carotid artery; iJV, internal jugular vein; eJV, external jugular vein, JLS, jugular lymph sac.

To further elucidate the role of *Gprc5b* in the development of lymphatics in mammals, we generated and characterized the phenotypes of *Gprc5b* global knockout mice (*Gprc5b^−/−^*). Following generation of the genetic mouse line (see Section 4—Materials and Methods), we used RNAscope to confirm the absence of *Gprc5b* transcripts in *Gprc5b^−/−^* mouse tissues, including within LECs of E14.5 embryos (Figure 3A, compared to Figure 1F).

Heterozygous *Gprc5b^+/−^* intercrosses were established and Mendelian ratios of live offspring at p10 for all genotypes did not differ significantly from the expected ratios (Pearson’s X^2^ = 3.77, *p* = 0.15). Embryos from timed matings were collected at E14.5—a developmental time point which follows the initial specification and formation of nascent lymphatic vessels (E9.5–E10.5), while still providing a time point of robust and stereotypic lymphangiogenesis within the thoracic region (see Figure 1A). At E14.5, compared to their wildtype *Gprc5b^+/+^* littermates, *Gprc5b^−/−^* embryos displayed a phenotypic range of subcutaneous edema, ranging from mild to severe (Figure 3B,C, yellow asterisks; *Gprc5b^+/+^*: zero embryos with edema of 13 total at e14.5; *Gprc5b^−/−^*: four embryos with edema of 16 total at e14.5; Chi-square value = 3.770, *p* = 0.0522). In addition, *Gprc5b^−/−^* embryos often showed small subcutaneous hemorrhagic plaques (Figure 3C, blue arrows), which were not observed among wildtype *Gprc5b^+/+^* littermates. Compared to the appearance of normal, intact tissue in *Gprc5b^+/+^* embryos (Figure 3D), histological analysis of the peripheral edema region confirmed fluid-filled and distended subcutaneous spaces in *Gprc5b^−/−^* embryos with both moderate and severe edema (Figure 3E,F, yellow asterisks), as well as subcutaneous hemorrhage in *Gprc5b^−/−^* embryos with severe edema (Figure 3F, blue arrows). Consistent with the interstitial edema and hemorrhage, we also observed the presence of pathological, blood-filled lymphatics in both the peripheral and thoracic areas of *Gprc5b^−/−^* embryos with severe edema (Figure 3F, blue arrows).

**Figure 4:**
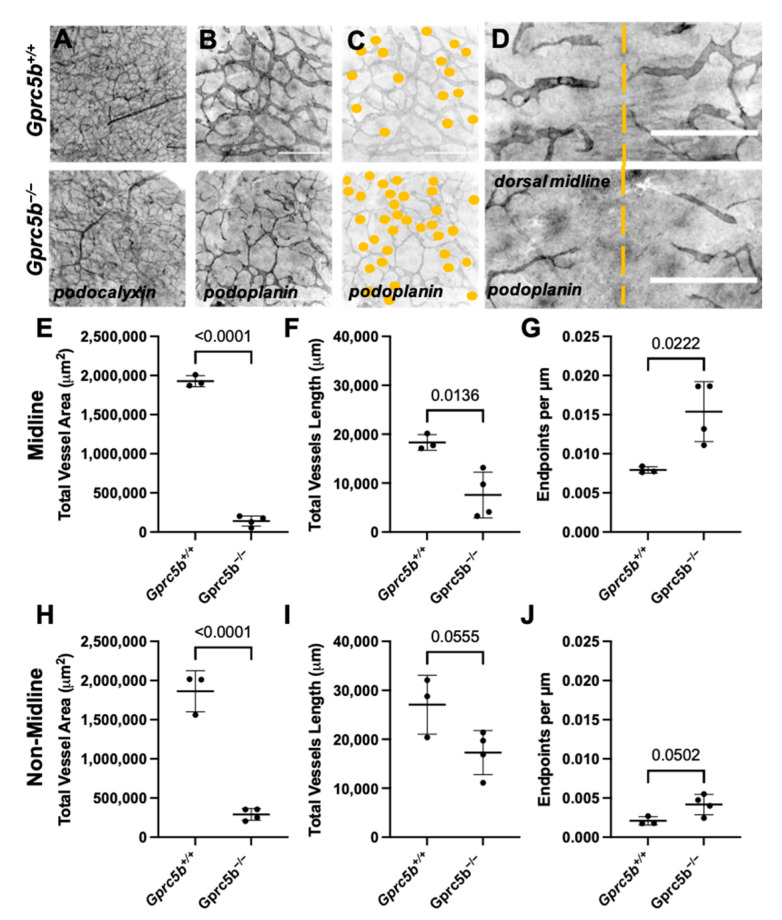
Histomorphometric analysis of vascular development reveals attenuated lymphangiogenesis in murine *Gprc5b^−/−^* embryos. Whole-mount immunostaining of dorsal skin from E14.5 *Gprc5b^+/+^* (top panels (**A**–**D**)) and *Gprc5b^−/−^* (bottom panels (**A**–**D**)) embryos, stained for the blood vascular marker podocalyxin (**A**) and the lymphatic marker podoplanin (**B**–**D**). Yellow dashed line indicates the dorsal midline (**D**). Scale bars = 500 µm (**E**–**J**). Quantification of total vessel area, total vessel length and relative endpoint counts of lymphatic vessels present at the dorsal back skin midline (**E**–**G**) and in the dorsal backskin in non-midline locations (**H**–**J**). Significance calculated using unpaired Student’s *t*-tests.

Whole mount immunostaining of the flank skin vasculature revealed normal, podocalyxin-positive blood vessels in *Gprc5b^−/−^* embryos compared to littermate control embryos (Figure 4A). However, the branching network of dermal lymphatic vasculature, identified as podoplanin-positive vessels, was markedly attenuated in E14.5 *Gprc5b^−/−^* embryos (Figure 4B). The lymphatic vessels of *Gprc5b^−/−^* mice appeared thin and whispy, with an incomplete network, as evidenced by the marked decrease in the total vessel area and total vessel length compared to *Gprc5b^+/+^* lymphatic vessels (Figure 4B–J). Additionally, the lymphatic vessels of *Gprc5b^−/−^* mice demonstrated an increased number of vessel endpoints per length of vessel, indicating a less dense network with more sprouts in *Gprc5b^−/−^* animals compared to wildtype (Figure 4C,G,J). In the dorsal back skin, lymphangiogenesis typically progressed toward the midline at E14.5, as evidenced by the close proximity of the lymphangiogenic fronts near the dorsal midline of *Gprc5b^+/+^* mice (Figure 4D, top panel). However, the growth and migration of dorsal lymphatics towards the midline was severely blunted by genetic loss of *Gprc5b* (Figure 4D, bottom panel, Figure 4D–G). The impaired lymphangiogenesis was also observed in non-midline dermal lymphatic vessels, which demonstrated reduced total lymphatic vessel area, length and endpoints per µm lymphatic vessel (Figure 4H–J).

Collectively, these data demonstrate that loss of *Gprc5b* in mice leads to embryonic edema and hemorrhage, associated with attenuated lymphangiogenesis characterized by reduced dermal network formation, reduced migration and thin lymphatic vessels. These pathological features are consistent with the developmental sequelae that characteristically occur with defective lymphangiogenesis and hydrops in both mice and humans.

### 2.5. Some Gprc5b^−/−^ Mice Exhibit Growth Restriction and Die Postnatally

**Figure 5:**
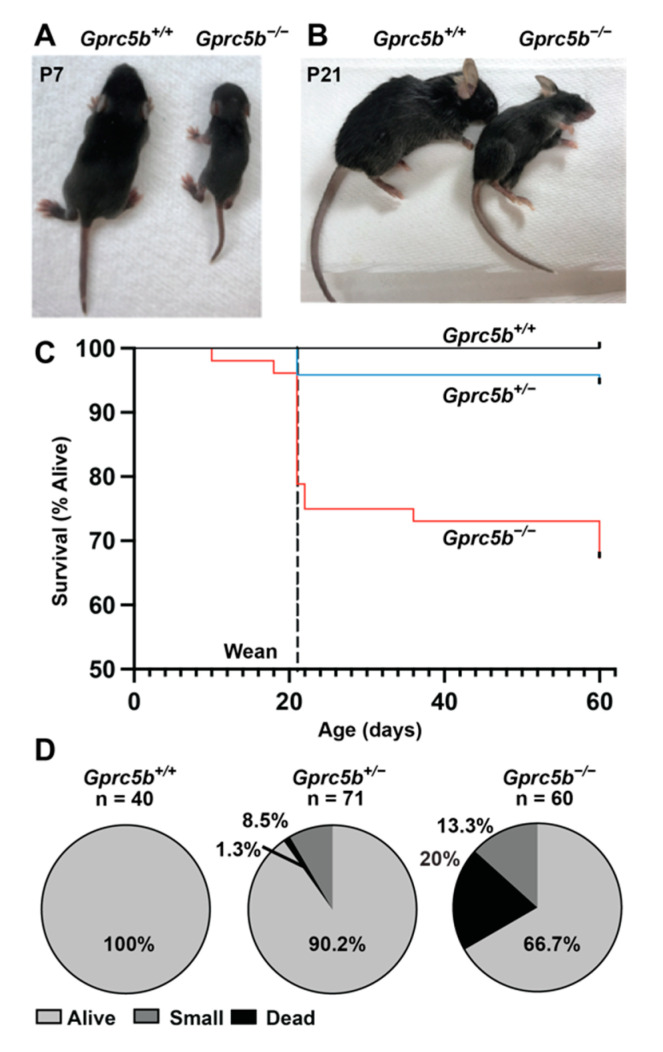
*Gprc5b^−/−^* mice die postnatally and exhibit growth restriction. (**A,B**) Representative images of *Gprc5b^+/+^* (left) and *Gprc5b^−/−^* (right) mice at postnatal days 7 (A) and 21 (B), showing marked growth restriction in *Gprc5b^−/−^* mice. (**C**) Survival curve shows ~25% loss of *Gprc5b^−/−^* mice prior to weaning. (**D**) Quantitation of growth restriction and death phenotypes by genotype from heterozygous *Gprc5b^+/−^* intercrosses.

We next sought to characterize the effects of Gprc5b loss on postnatal and adult lymphatics. However, during the breeding of *Gprc5b^+/−^* mice, we noticed that many *Gprc5b^−/−^* mice appeared small and growth-restricted compared to their *Gprc5b^+/+^* littermates at postnatal day 7 (Figure 5A) and postnatal day 21 (Figure 5B), similar to results previously reported for an independently generated *Gprc5b* global knockout mouse line [26]. Indeed, by weaning age we quantified an approximately 20–25% early death rate for *Gprc5b^−/−^* mice (Figure 5C), with an additional 13% exhibiting growth restriction (Figure 5D). This is congruent with the embryonic data, in which 25% of e14.5 *Gprc5b^−/−^* embryos demonstrated overt edematous phenotypes (Figure 3C). A small number of *Gprc5b^+/−^* mice (N = 6 out of 71) also exhibited growth restriction, while all *Gprc5b^+/+^* mice (N = 40) survived and had no remarkable pathology. Moreover, the surviving non-growth-restricted *Gprc5b^−/−^* mice had a similar body size and life span compared to their *Gprc5b^+/+^* and *Gprc5b^+/−^* littermates. These data indicate that global deficiency, as well as haploinsufficiency, for *Gprc5b* may be associated with a failure to thrive or increased mortality.

### 2.6. Gprc5b^−/−^ Mice Exhibit Attenuated Postnatal Lymphatic Growth

**Figure 6:**
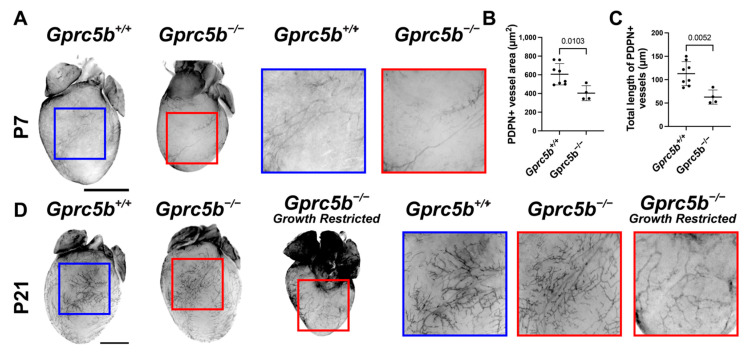
Attenuated postnatal cardiac lymphangiogenesis in developing and growth-restricted *Gprc5b^−/−^* mice. (**A**) Whole-mount immunostaining of podoplanin (PDPN)-positive cardiac lymphatics of P7 *Gprc5b^+/+^* hearts (left) and *Gprc5b^−/−^* hearts (right). (**B**) Quantification of PDPN-positive vessel area of dorsal or ventral surface of the heart. (**C**) Quantification of total PDPN-positive vessel length of dorsal or ventral surface of the heart. Significance calculated using unpaired *t*-test. (**D**) Whole-mount immunostaining of podoplanin-positive cardiac lymphatics of P21 *Gprc5b^+/+^* hearts (left), non-growth-restricted (middle) and growth-restricted (right) *Gprc5b^−/−^* hearts. High magnification of insets provided to the right of full heart images. Blue border = *Gprc5b^+/+^*, red border = *Gprc5b^−/−^*. Scale bar = 2 mm.

The growth and maturation of the lymphatic vascular system continues in many organ beds during the postnatal period. For example, cardiac lymphatics robustly grow and expand to cover the surface of the myocardium between postnatal day 1 and postnatal day 15 [27]. Interestingly, we found that cardiac lymphatic growth and surface area coverage, as visualized by podoplanin whole-mount immunostaining of the heart, was significantly attenuated on postnatal day 7 in growth-restricted *Gprc5b^−/−^* mice compared to *Gprc5b^+/+^* controls (Figure 6A–C). By postnatal day 21, the *Gprc5b^−/−^* mice that did not suffer from growth restriction exhibited normal and robust cardiac lymphangiogenesis (Figure 6D). However, cardiac lymphangiogenesis was persistently attenuated in the growth-restricted *Gprc5b^−/−^* animals (Figure 6D), despite their equivalent heart weight:body weight ratios (*Gprc5b^+/+^* = 5.467 mg/g +/− 0.15 SEM; *Gprc5b^−/−^* = 5.53 mg/g +/− 0.12 SEM; *n* = 3/group, *p* = 0.8). Together, these data indicate that expression of *Gprc5b* during the postnatal period is important during the development of normal organ-specific lymphangiogenesis in mammal hearts.

### 2.7. GPRC5B Deficiency Reduces Human LEC Proliferation and Viability In Vitro

To elucidate the cellular effects of *GPRC5B* deficiency in lymphatic endothelial cells and establish its functional role in human cells, *hGPRC5B* was knocked-down in primary cultured human LECs using two independent lentivirus-delivered shRNA sequences. *GPRC5B* shRNA knockdown efficiency was determined by qPCR where *GPRC5B* expression levels in shRNA-treated conditions were normalized to untreated LEC controls. *GPRC5B* transcript expression in non-targeting *shHBG*-treated LECs was not significantly different from the expression of *GPRC5B* in untreated LECs (*shHBG* = 1.658 +/− 0.92 SEM, unpaired Student’s *t*-test with Welch’s correction *p* = 0.5585). *GPRC5B* expression in LECs treated with either of the *GPRC5B*-targeting shRNA clones was significantly reduced by over 90% compared to untreated LECs (*shGPRC5B-1* = 0.083 +/− 0.045 SEM compared to untreated LECs *p* = 0.011; *shGPRC5B-2* = 0.088 +/− 0.029 SEM compared to untreated LECs *p* = 0.015, unpaired Student’s *t*-test with Welch’s correction).

**Figure 7:**
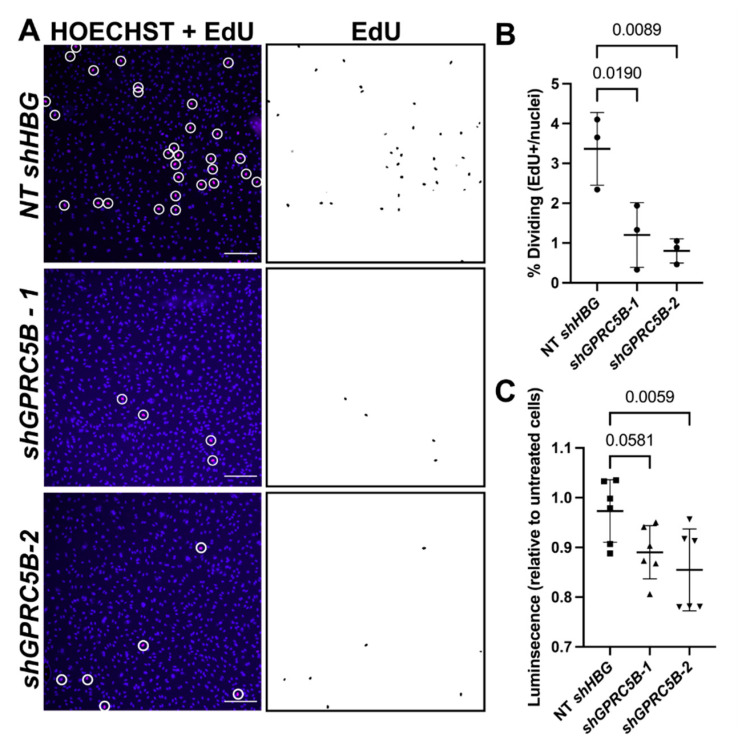
*hGPRC5B*-deficient human lymphatic endothelial cells are less proliferative and less viable compared to control cells. (**A**) Immunofluorescence of human dermal lymphatic endothelial cells treated in vitro with non-targeting control shRNA or shRNA targeting *GPRC5B*. Left column: red puncta encircled in white indicate Edu-positive cells. Right column: monostaining of EdU-positive nuclei. (**B**) Quantification of EdU-positive lymphatic endothelial cells. (**C**) CellTiter-Glo cell viability assay where luminescence indicates viable cells. Significance calculated using one-way ANOVA with correction for multiple comparisons. Scale bar = 50 µm.

Following successful shRNA knockdown of *GPRC5B*, basal LEC proliferation and viability were evaluated. The percent of EdU positive cells (number of EdU-positive nuclei divided by number of HOECHST-positive nuclei) was significantly reduced in LECs that were deficient in *GPRC5B* (Figure 7A,B). Additionally, cultured hLECs that were deficient in *GPRC5B* were also less viable than untreated LECs or LECs that were treated with control shRNA (Figure 7C). Collectively these data demonstrate that maintained expression of *GPRC5B* is required for the viability and proliferation of cultured primary human LECs.

## 3. Discussion

The relatively understudied lymphatic vascular system lacks precise pharmacological tools. However, lymphatics are implicated in development and in many disease conditions, such as primary lymphedema, lymphatic vascular malformations, cancer metastasis, inflammation, gastrointestinal malabsorption and organ transplant rejection. Therefore, it is important to explore the molecular and cellular targets that govern lymphatic endothelial cell specification, proliferation, migration, permeability and function. Here, we focused on illuminating GPCRs that have enriched expression in human lymphatic endothelial cells compared to human blood endothelial cells. We further refined our focus to four orphan receptors, GPRC5B, ADGRF5/GPR116, FZD8 and GPR61, because three of the four showed the highest levels of expression among all identified targets. Similar strategies have been previously used with ex vivo mouse endothelial cells, resulting in the identification of several additional orphan adhesion family receptors (ADGRG3/Gpr97, ADGRA2/Gpr124) and a family member of our highest-expressed receptor, *Gprc5C* [28,29]. It is interesting to speculate, but we consider that the preponderance of orphan receptors at the top of lists for lymphatic-enriched GPCRs may be inextricably concomitant with the relatively understudied nature of lymphatics.

GPCR class C group 5 member B (GPRC5B) is an orphan GPCR that belongs to family C of the GPCRs. It is highly expressed in the brain [29,30], adipose tissue [31] and kidney [32,33]. In an independently generated global knockout mouse line, *Gprc5b^−/−^* mice showed behavioral abnormalities [26], morphological abnormalities in the Purkinje cell axons and disruption of cerebellar synaptic plasticity and motor learning [34]. Additionally, *Gprc5b* also modulates inflammatory responses in adipocytes [31], cardiac fibroblasts [35] and kidney glomeruli [33]. Consistent with the growth restriction and failure to thrive which we report here, prior studies also showed that loss of *Gprc5b* protected mice from diet-induced obesity and insulin resistance due to decreased inflammation in white adipose tissue [31]. Moreover, dozens of genome-wide association studies have linked variants in *GPRC5B* with altered body mass index [36,37]. The extent to which the expression and function of GPRC5B in postnatal lymphatics contributes to these physiological effects remains an area for further exploration.

The role of *Gprc5b* in vascular systems, particularly in the lymphatic vasculature, has not been well-characterized. Using both zebrafish and murine models, we consistently found that genetic loss of *Gprc5b* caused attenuated lymphatic growth during embryogenesis. Moreover, knockdown of *GPRC5B* from cultured primary human LECs also attenuated their proliferation and viability, supporting an essential function for Gprc5b in lymphatic maintenance across species. Reduced proliferation markers were also observed in Gprc5b-deficient smooth muscle cells, attributable in part to the inhibition of the G_s_-coupled prostacyclin receptor (IP), which has anti-proliferative effects [38]. In other studies, *Gprc5b* expression was associated with enhanced β cell proliferation [39] and neurogenesis [40]. Collectively, these studies support a pro-proliferative role for *Gprc5b* in many cell types including LECs and suggest that future high-throughput ligand screening for this orphan receptor may be valuable.

## 4. Materials and Methods

### 4.1. GPCR Pathway Focused Array

This work was performed by the Duke University DNA Analysis Facility using Human GPCR TaqMan Array Micro Fluidic Cards (Applied Biosystems, Bedford, MA, USA, cat. 4367785) inclusive of 380 non-odorant GPCRs, with a Quantstudio 12K flex real-time instrument used for RNA isolated from primary human umbilical venous endothelial cells (HUVECs; Lonza CC-2517) and primary dermal lymphatic endothelial cells (LECs; Lonza CC-2812). Experimental protocols were followed according to the manufacturer’s instructions. *n* = 4 for each cell type. Data were analyzed using Applied Biosystems 7900HT software (cat 4365295).

### 4.2. RNAscope and Immunohistochemistry

RNAscope (ACD Bio) was performed prior to immunohistochemistry. First, 5 µm paraffin sections were deparaffinized, hydrated, and permeabilized. Samples were then treated at room temperature with hydrogen peroxide. Antigen retrieval was then performed by incubating slides in ACD Bio target retrieval buffer for 15 min at 100 °C. Samples were then allowed to dry overnight. The following day samples were treated with ACD Bio Proteinase Plus for 30 min. Samples were then incubated with positive, negative and GPCR-specific targeting probes for 2 h. Probes were amplified using ACD Bio probe signal amplifying reagents and were conjugated to cyanine 5 fluorophore (monostaining) or cyanine 3 fluorophore (for a second probe channel). Immunofluorescence was then performed on the samples. Samples were blocked with 5% normal donkey serum in PBS and then incubated with primary antibody goat anti-LYVE1 (1:500, R&D Systems, Minneapolis, MN, USA, AF2125-SP) overnight at 4 °C. Sections were rinsed with PBS with 5% normal donkey serum three times for 5 min. Samples were then incubated with secondary antibody donkey anti-goat IgG AF488 (Jackson ImmunoResearch Laboratories, West Grove, PA, USA, 705-545-003) in the dark for 2 h at room temperature. Slides were incubated with Hoechst 33258 (1:1000, Sigma Aldrich, St. Louis, MO, USA) for 10 min at room temperature. Samples were mounted with ProLong Gold Antifade Mounting Media (Thermo Fisher Scientific, Waltham, MA, USA). Images were captured on a Nikon Eclipse E800 microscope with a Hamamatsu ORCA-ER camera.

### 4.3. Animals

All protocols using animals were approved by the Institutional Animal Care and Use Committee of the University of North Carolina at Chapel Hill.

#### 4.3.1. Zebrafish

AB *Tg(fli1a:EGFP)^y1^* [41] *Tg(kdrl:Hsa.HRASmCherry)^s896^* [42] fish were used in this study. Embryos were staged by hour post-fertilization at 28.5 °C. MOs (*gprc5ba* MO (1 ng)) were injected into one-cell stage embryos. The sequences of MOs used were as follows: *gprc5b* MO, 5′- CACATCTGAGGACACAAGAGGACAG-3′) (Gene Tools). Pigmentation of embryos and larvae was inhibited by 1-phenyl-2-thiourea (Sigma). The embryos were treated with 100 mg/mL tricaine (Sigma), mounted in a drop of 1.0–1.5% low-melting agarose in egg water and placed onto a glass-bottom Petri dish (MatTek Corporation, Ashland, MA, USA). Fluorescence images were obtained using an LSM800 confocal laser scanning microscope (Zeiss, Jena, Germany), an Olympus Fluoview FV1000 confocal laser scanning microscope (Olympus, Tokyo, Japan) or Nikon SMZ25 high-end stereoscopic microscope (Nikon, Melville, NY, USA). Three-dimensionally-rendered z-stack images and three-dimensional surface-rendered images and movies were analyzed and assembled using the IMARIS software (BITPLANE).

#### 4.3.2. Mice

To generate *Gpcr5b* knockout mice, *Gprc5b^tm1a(EUCOMM)Wtsi^* ES cells (C57BL/6NTac) obtained from the European Mouse Mutant Cell Repository (EuMMCR) were injected into blastocysts to generate *Gprc5b^tm1a/+^* heterozygous mice. *Gprc5b^tm1a/+^* heterozygous mice were then crossed to a β-actin cre transgenic line to delete exon 2, which contains the ATG start codon of *Gprc5b*, resulting in *Gprc5b^tm1b/+^* mice, herein also called *Gprc5^+/−^*. *Gprc5^+/−^* mice were then backcrossed for at least ten generations onto a C57BL6/NCr background. For experimental assays, *Gprc5^+/−^* mice were intercrossed to generate wildtype *Gprc5b^+/+^* or *Gprc5b^−/−^* knockout mice. Littermates were used for control experiments.

### 4.4. Histology

Mouse embryos were dissected at E14.5, fixed in 4% paraformaldehyde overnight and embedded in paraffin for sectioning. Then, 5 μm paraffin sections were obtained for haemotoxylin and eosin (H&E) staining. Sectioning and H&E staining were performed by the UNC Histology Research Core Facility.

### 4.5. Whole-Mount Immunostaining

Embryonic back skin was collected at E14.5. Hearts from either the neonatal period or age of weaning were dissected. Tissues were rinsed in PBS and fixed in 4% PFA overnight at 4 °C. Whole-mount immunostaining as was performed as previously reported with slight changes [43]. Tissues were washed 5 min in PBST (0.1% TritonX-100), 5 min in H_2_O, 7 min in acetone at −20 °C, 5 min in H_2_O and 5 min in PBST and then blocked in blocking solution (5% donkey serum, 0.1% BSA and 0.1% DMSO in PBST) at 4 °C overnight. Primary antibodies were applied (anti-podoplanin, Syrian Hamster; Developmental Studies Hybridoma Bank, 1:200) for 5 days, then tissues were washed in washing solution (0.1% BSA, 0.1% DMSO in PBST) for 4 × 10 min, blocked in the blocking solution at room temperature for 1.5 h and incubated in AffiniPure Rabbit Anti-Syrian Hamster IgG 647 (1:200 in blocking solution) 48 h at 4 °C. The tissues then were washed in the washing solution for 5 × 10 min and preserved in PBS. The skin was mounted on slides with ProLong Gold Antifade Mounting Media (Thermo Fisher Scientific) and imaged using an I83 Olympus inverted fluorescence microscope with a Hamamatsu camera. The heart was imaged using a Leica MZ16FA (Hooker Imaging Core, UNC-Chapel Hill). AngioTool [44] was used for quantification.

### 4.6. Cell Culture

Primary human dermal lymphatic endothelial cells, isolated from juvenile human foreskin (LECs, PromoCell, C-12216), were used within five passages and cultured in an Endothelial Cell Growth Medium MV2 kit (PromoCell, C-22121) at 37 °C under 5% CO_2_.

### 4.7. Lentivirus-Mediated Knockdown of GPRC5B in LECs

Lentiviral particles containing small hairpin RNA constructs designed to specifically target *GPRC5B* (*shGPRC5B*, oligo 1 sequence: CCGGCGCAAACTAAAGCAAAGCTAACTCGAGTTAGCTTTGCTTTAGTTTGCGTTTTTG; oligo 2 sequence: CCGGCCGTTTAGAAGCAACGTGTATCTCGAGATACACGTTGCTTCTAAACGGTTTTTG) or a control shRNA that targets human beta globin (*shHBG*, oligo sequence: CCGGCCCATCACTTTGGCAAAGAATCTCGAGATTCTTTGCCAAAGTGATGGGTT-TTTG), a gene for which there should be no mRNA present in LECs, were used. The lentivirus was generated by the UNC Lenti-shRNA Core Facility. LECs were plated and allowed to grow for 24 h. Cells were transduced with either *shHBG* or *shGPRC5B* lentivirus. Media were changed the next day, and then allowed to rest for an additional day. To confirm effective knockdown, LECs transduced with lentivirus were harvested with TRIzol Reagent (Invitrogen, Thermo Fisher Scientific). Reverse transcription was performed using M-MLV reverse transcriptase (Thermo Fisher Scientific, Cat. No. 28-025-013). Quantitative gene expression was assessed using the TaqMan gene expression paradigm (Applied Biosystems) and PCR was performed using a Quant Studio 7 Flex Real-Time PCR System (Applied Biosystems). The following Thermo Fisher Taqman gene expression assay probes were used for real-time PCR (qPCR): *GPRC5B-Hs00212116_m1* and *GAPDH-Hs02786624_g1*. Relative expression levels were determined with the ΔΔCt method and normalized to reference gene expression of *GAPDH*.

### 4.8. EdU Cell Proliferation Assay

LECs transduced with either *shHBG* or *shGPRC5B* lentivirus were incubated with 10 µM EdU (5-ethynyl-2′-deoxyuridine) for 4 h. EdU staining was performed based on the instruction of the Click-iT™ EdU Cell Proliferation Kit for Imaging (Thermo Fisher Scientific, C10339). Cells were rinsed with PBS and then fixed with 4% PFA for 30 min. Cells were then rinsed three times for 5 min with PBS, then permeabilized in 0.5% Triton X-100 for 15 min followed by three 5 min washes in PBS with 3% Bovine Serum Albumin. Then, each well was incubated with 500 µL of the EdU kit (Invitrogen, Waltham, MA, USA, C10339) for 30 min at room temperature protected from light. Cells were then washed once with 3% Bovine Serum Albumin in PBS and incubated with HOECHST 33258 (1:1000, Sigma Aldrich) for 10 min. Cells were imaged using an Olympus IX83 inverted microscope and images taken with a Hamamtsu ORCA Flash 4.0 camera. Percent EdU-positive cell ratio was determined by the number of EdU-positive nuclei divided by the number of HOECHST-positive nuclei.

### 4.9. Viability Quantification Using CellTiter-Glo

LECs were removed from the incubator and allowed to equilibrate to room temperature for 30 min. Then, 100 µL of CellTiter-Glo (Promega, Madison, WI, USA, G7570) reagent was added to the existing cell culture media. The plate was then placed on an orbital shaker to induce lysis. The cells were then allowed to incubate at room temperature for 10 min to allow for the luminescence to stabilize. Then, luminescence was recorded using a Mithras LB 940 Multimode Microplate Reader (Berthold Technologies, Vienna, Austria).

## 5. Conclusions

The need for the identification of novel lymphatic-enriched drug targets is immense. Although future studies are required to delineate the key endogenous ligands and downstream signaling pathways of GPRC5B, the present study underscores the importance of this orphan receptor in the development of lymphatics and lays the foundation for exploring the utility of this and other lymphatic-enriched orphan GPCRs as potential therapeutic targets for lymphatic disorders.

## Figures and Tables

**Table 1:** Significantly upregulated GPCRs in human dermal lymphatic endothelial cells.

Target ID	Gene Symbol	Receptor Protein Name	*p*-Value	Log_2_ Fold Change
GPRC5B-Hs00212116_m1	***GPRC5B***	GPCR Receptor Class C Group 5 Member B **(Orphan)**	0.002	11.83
GPR116-Hs00391810_m1	***ADGRF5***	Adhesion G Protein-Coupled Receptor F5 **(Orphan)**	0.010	6.75
FZD8-Hs00259040_s1	***FZD8***	Frizzled Class Receptor 8 **(Orphan)**	0.019	3.10
EDG1-Hs00173499_m1	*S1PR1*	Sphingosine-1-Phosphate Receptor 1	0.033	2.87
SSTR5-Hs00265647_s1	*SSTR5*	Somatostatin Receptor 5	0.036	2.10
FKSG83-Hs00259293_s1	*VN1R10P*	Vomeronasal 1 Receptor 10 Pseudogene	0.041	1.85
GPR10-Hs00244685_s1	*PRLHR*	Prolactin-releasing peptide receptor	0.049	1.82
PPYR1-Hs00275980_s1	*NPY4R*	Neuropeptide Y Receptor Y4; Pancreatic Polypeptide Y	0.008	1.71
GPR55-Hs00271662_s1	*GPR55*	G Protein-Coupled Receptor 55	0.010	1.64
CHRM2-Hs00265208_s1	*CHRM2*	Cholinergic Receptor Muscarinic 2	0.011	1.46
MC3R-Hs00252036_s1	*MC3R*	Melanocortin 3 Receptor	0.043	1.37
HTR1F-Hs00265296_s1	*HTR1F*	Serotonin Receptor 1F	0041	1.35
GPR61-Hs00259108_s1	***GPR61***	G Protein-Coupled Receptor 61 **(Orphan)**	0.046	1.34
VN1R2-Hs00545195_s1	*VN1R2*	Vomeronasal Type-1 Receptor 2, GPCR25	0.036	1.33
ADRB2-Hs00240532_s1	*ADRB2*	Beta-2 Adrenergic Receptor	0.047	1.31
GPR120-Hs00664157_s1	*FFAR4*	Free Fatty Acid Receptor 4	0.032	1.25
OR2C3-Hs00812303_s1	*OR2C3*	Olfactory Receptor Family 2 Subfamily C Member 3	0.040	1.24

Log_2_ mean intensity compared to human umbilical venous endothelial cells, *p* < 0.05, fold-change > 1.2. Bolded gene symbol indicates an orphan GPCR.
